# Evaluating the Frequency of Osmophobia in Tension-Type Headache: A Comparative Study on Migraine

**DOI:** 10.7759/cureus.66558

**Published:** 2024-08-10

**Authors:** Esen Çiçekli, Semra Ozturk Mungan, Gürdal Orhan

**Affiliations:** 1 Neurology Department, Akyazı State Hospital, Sakarya, TUR; 2 Neurology Department, Ankara City Hospital, Ankara, TUR

**Keywords:** vas, midas, osmophobia, tension-type headache, migraine

## Abstract

Introduction

Osmophobia is hypersensitivity to certain odors. Although osmophobia is a symptom related to migraine, it is also reported in tension-type headache (TTH). Osmophobia is recommended for inclusion in the migraine diagnostic criteria because it increases sensitivity and shows absolute specificity. However, there is no evidence of the association between TTH and osmophobia. This study aimed to evaluate the prevalence and clinical characteristics of osmophobia in a cohort of migraine and TTH patients.

Methods

For the current analysis, patients who met the inclusion criteria among patients diagnosed with migraine and TTH according to the International Classification of Headache Disorders III criteria in the neurology outpatient clinics of Ankara City Hospital and Akyazı State Hospital were selected retrospectively. A total of 214 patients (129 with migraine and 85 with TTH) were included in the study. Patients’ characteristics, visual analog scale (VAS) pain scores, and Migraine Disability Assessment Scale (MIDAS) scores were recorded. Osmophobia characteristics in migraine and TTH patients were compared along with clinical parameters between the groups and within the groups.

Results

Osmophobia was found in 68% of migraine patients. The most common type of smell that migraine patients experienced was the scent of perfume. A total of 31.3% of the patients with TTH had osmophobia. While the most irritating odorant in migraine patients was perfume (32%), in TTH patients, it was the smell of food (10.5%). There were no significant differences between osmophobia, and age, education level, disease duration, pain frequency, attack duration, or VAS score in both migraine patients and TTH patients. There was also no significant difference between migraine patients with (2.42) and without (2.33) osmophobia in terms of the MIDAS score.

Discussion

Our study indicates that osmophobia observed in migraine is valuable in differential diagnosis. However, it can be significantly identified in TTH patients. It should be used together with other supporting criteria in differential diagnosis. It would also be useful to question the characteristics of osmophobia in more detail in the anamnesis.

## Introduction

The sense of smell abnormalities has been associated with many diseases, especially neurodegenerative diseases, for many years [1]. Osmophobia can be defined as increased sensitivity to specific odors. It is an isolated symptom that is especially common in patients with primary headaches [2,3]. Osmophobia in primary headaches has been reported at different frequencies in studies. In clinical practice, the presence of osmophobia more often directs the clinician to the diagnosis of migraine [2]. These individuals may also exhibit increased odor sensitivity compared to normal individuals during the period between attacks. Whether there is a change in odor perception thresholds in migraine patients during the interictal period is controversial [2,4].

A new headache is coded as a primary headache if it does not occur in a close temporal relationship with another disorder known to cause the headache or if it does not meet other criteria for causality of that disorder [5]. There are no specific criteria for diagnosing primary headaches; however, the diagnosis is mainly supported by anamnesis. Laboratory and imaging methods are only used to exclude secondary causes of headaches [3].

It is recommended that osmophobia be included in the migraine diagnostic criteria because it increases sensitivity, that is, the rate of detecting real migraine patients among patients, and shows absolute specificity, that is, its false-positive rate is low [2]. Osmophobia is more common in patients with a longer duration of migraine and more migraine-related disorders. These results suggest that sensitivity increases with increasing migraine burden [6,7]. Although the relationship between migraine and osmophobia is not clear, connections between the olfactory system and the trigeminal nociceptive system have been emphasized [8,9].

Although there are publications supporting the frequency of osmophobia in individuals with tension-type headache (TTH), the association between TTH and osmophobia has not been definitively demonstrated, and various studies have been conducted on this subject [1,10]. Osmophobia is also present in chronic TTH patients. It has also been associated with central sensitization symptoms such as allodynia [3]. Considering this information, other studies have stated that criteria other than osmophobia are required for the differential diagnosis of migraine and TTH [11].

Factors such as the duration of the attack, the similarity of accompanying symptoms, and ability of the TTH patient to describe photophobia-phonophobia may make differential diagnosis difficult. For this reason, we believe that symptoms attributed to migraine, such as osmophobia, should be studied frequently and in different populations to increase supporting evidence. This study aimed to evaluate the prevalence and clinical characteristics of osmophobia in a cohort of migraine and TTH patients selected from two different centers. With the data obtained, we aimed to overcome the difficulties in the differential diagnosis of primary headache patients, determine the relationship between osmophobia and primary headache or severe clinical presentation, evaluate the guiding role of osmophobia in initiating preventive treatment, and contribute to the literature with our own series.

This article was previously posted to the Research Gate preprint server in May 2024.

## Materials and methods

This retrospective study was based on data collected from the neurology outpatient clinics of Akyazı State Hospital and Ankara City Hospital. This study was approved by the Ankara City Hospital Medical Research Scientific and Ethical Evaluation Board (number 2-24-51). Written informed consent was obtained from all the participants.

Patient selection

Current data were obtained from the hospital electronic database of migraine and TTH patients who visited the neurology outpatient clinics of Akyazı State Hospital and Ankara City Hospital between January 1, 2023, and January 1, 2024. The characteristics of the patients, such as age, gender, headache type, disease duration, duration of attacks, attack frequency, comorbidities, and medications, were recorded for retrospective analysis. For the current analysis, patients aged between 18 and 65 years who presented to the neurology outpatient clinic and were diagnosed with migraine or TTH were selected according to the International Classification of Headache Disorders III [5]. Patients with serious comorbidities (such as liver, kidney, and cardiovascular failure), previous or current neurological diseases other than migraine, current or previous psychiatric diseases, or any disease that could cause olfactory disorders were excluded from the study.

Patient evaluation

Patient data were recorded, including demographic information, headache type, and pain characteristics. Patients with daily or near-daily headache resulting from overuse of one or more migraine headache-relieving analgesic drug classes were considered to have analgesic abuse. Previous studies have shown that patients were administered an oral semi-structured questionnaire to assess the presence of osmophobia [1]. In our study, the presence and type of osmophobia were recorded from the patient's past anamnesis. Pain intensity was assessed using the visual analog scale (VAS) pain score. For this evaluation, the patient was asked to indicate the severity of his pain on a scale of 0 to 10. The presence or absence of osmophobia during the attack and inter-ictal periods was recorded using anamnesis information. Pain-related disability in patients with migraine was evaluated using the Migraine Disability Assessment Scale (MIDAS) [12], and the patients were divided into four subgroups according to severity (Table 1).

**Table 1:** MIDAS classification MIDAS, Migraine Disability Assessment Scale

MIDAS score	Disability	MIDAS grade
0-5	Little or no disability	I
6-10	Mild disability	II
11-20	Moderate disability	III
21+	Severe disability	IV

Statistical analysis

Statistical analysis of the data was performed using SPSS Version 23 (IBM Corp., Armonk, NY). The data were normally distributed because the skewness and kurtosis values were between -1.5 and +1.5. Since the data were normally distributed, the significance of the difference between groups was tested using an independent t-test. The significance of the difference in dichotomous data between groups was evaluated using the chi-square test. The significance of the differences between more than two groups was tested using ANOVA. Because the groups were not distributed homogeneously according to the Levene test, the Games-Howell test was used as a post hoc test.

## Results

A total of 214 patients (128 with migraine and 86 with TTH) were included in the study. The mean age of the TTH patients was greater than that of the migraine patients (p<0.05). There was no significant difference between migraine patients and TTH patients in terms of education level or years of pain (p>0.05). The frequency of pain attacks in TTH patients within one month was greater than that in migraine patients (p<0.05). The attack duration and VAS score were greater in patients with migraine than in those with TTH (p<0.05) (Table 2).

**Table 2:** Age, education level, disease duration, pain frequency, attack duration, and VAS score characteristics of the migraine and TTH groups TTH, tension-type headache; VAS, visual analog scale

Characteristics of the patients	Headache type	Mean ± SD	P-value
Age (years)	Migraine	36.26±9.64	<0.001
	TTH	43.07±13.00	
Education level (primary, high school, college)	Migraine	2.39±1.26	0.64
	TTH	2.31±1.13	
Disease duration (years)	Migraine	8.37±8.07	0.218
	TTH	7.07±6.76	
Pain frequency (in a month)	Migraine	4.49±3.52	<0.001
	TTH	6.91±3.35	
Attack duration (hours)	Migraine	42.03±25.35	<0.001
	TTH	13.05±14.24	
VAS score (1-10)	Migraine	8.10±1.56	<0.001
	TTH	6.84±1.34	

The number of women was higher than the number of men in the migraine and TTH groups (P<0.05) (Table 3).

**Table 3:** Gender distribution in the migraine and TTH groups TTH, tension-type headache

Migraine				TTH		
	N	%	P-value	N	%	P-value
Female	108	83.7	0.01	60	70.6	0.02
Male	21	16.3		25	29.4	
Total	129	100		85	100	

There was no relationship between gender and osmophobia in migraine and TTH patients (p>0.05) (Table 4).

**Table 4:** The relationship between osmophobia and gender in the migraine and TTH groups TTH, tension-type headache

	Migraine				TTH			
	Osmophobia				Osmophobia			
	Present	Absent	Total	P-value	Present	Absent	Total	P-value
Female	75	33	108	0.391	16	44	60	0.797
Male	12	9	21		6	19	25	
Total	87	42	129		22	63	85	

Osmophobia was more common in migraine patients than in TTH patients, and the frequency of osmophobia was also greater during the inter-ictal period in migraine patients (p<0.05). There was no statistically significant difference in analgesic abuse between the migraine and TTH groups (p>0.05) (Table 5).

**Table 5:** Osmophobia and analgesic abuse frequency in migraine and TTH patients. TTH, tension-type headache

Clinical parameter	Headache type	N	P-value
Presence of osmophobia	Migraine	87	<0.001
	TTH	22	
	Total	109	
Osmophobia in the inter-ictal period	Migraine	40	<0.001
	TTH	10	
	Total	50	
Analgesic abuse	Migraine	41	0.581
	TTH	24	
	Total	65	

Overall, 68% of the patients with migraine had osmophobia. The most common type of smell that migraine patients experienced was the scent of perfume. The smell of the perfume was followed by the smell of food, cigarettes, and sweat. A total of 31.3% of the patients with TTH had osmophobia. The odor of TTH patients was the most disturbed by the smell of food, followed by the smell of cigarettes and perfumes (Table 6).

**Table 6:** Type of odors that disturbed migraine and TTH patients TTH, tension-type headache

Type of odorant	Migraine		TTH	
	N	%	N	%
Perfume	40	32	5	5.8
Detergent	6	4.7	3	3.5
Cigarettes	8	6.3	7	8.2
Bleach	2	1.6	1	1.1
Food	19	14.8	9	10.5
Coffee	1	0.8	0	0
Onion-garlic	2	1.6	1	1.1
Sweat	7	5.4	0	0
Spices	1	0.8	1	1.1
Non	40	32	59	68.7
Total	128	100	86	100

There were no significant differences in osmophobia or age, education level, disease duration, pain frequency, attack duration, or VAS score between migraine patients and TTH patients (p>0.05 in all). There was no statistically significant difference between migraine patients with and without osmophobia in terms of the MIDAS score (p>0.05) (Table 7).

**Table 7:** Relationship of osmophobia with age, education level, disease duration, pain frequency, pain duration, and VAS scores in migraine and TTH patients and of osmophobia with MIDAS scores in migraine patients TTH, tension-type headache; VAS, visual analog scale; MIDAS, Migraine Disability Assessment Scale

Characteristics of the patients	Osmophobia	Migraine		TTH	
		Mean ± SD	P-value	Mean ± SD	P-value
Age (years)	Present	35.12±9.14	0.053	40.90±12.96	0.368
	Absent	38.61±10.30		43.82±13.04	
Education level (primary, high school, college)	Present	2.50±1.24	0.603	2.50±1.10	0.417
	Absent	2.38±1.32		2.26±1.15	
Disease duration (years)	Present	7.72±8.02	0.189	6.00±7.13	0.392
	Absent	9.72±8.10		7.44±6.64	
Pain frequency (in a month)	Present	4.37±3.50	0.590	6.50±3.50	0.488
	Absent	4.73±3.60		7.06±3.18	
Pain duration (hours)	Present	40.63±23.92	0.369	13.18±16.61	0.963
	Absent	44.92±28.16		13.01±13.47	
VAS score (1-10)	Present	8.13±1.62	0.760	6.63±1.25	0.395
	Absent	8.04±1.44		6.92±1.37	
MIDAS score (1-4)	Present	2.42±1.04	0.645		
	Absent	2.33±1.09			

VAS and MIDAS scores were greater in migraine patients with analgesic abuse than in those without. The educational level of migraine patients with analgesic abuse was lower than that of those without abuse (p<0.05). The mean age, disease duration, pain frequency, and mean VAS score of TTH patients with analgesic abuse were greater than those of patients without analgesic abuse (p<0.05). There was no statistically significant relationship between osmophobia and analgesic abuse in patients with migraine or TTH (p>0.05).

## Discussion

In our study, 68% of patients with migraine had osmophobia. The frequency of osmophobia in migraine patients is reported to be between 25% and 95% in recent studies [2,13,14]. Our findings regarding the migraine and osmophobia relationship support the literature.

Some studies investigating osmophobia during attacks have reported that osmophobia is observed only in migraine patients and not in TTH patients, and it has been concluded that osmophobia during attacks is specific to migraines [1,2]. In our study, although there were TTH patients who experienced osmophobia both during attacks and during the inter-ictal period, the number of these patients was significantly lower than that of migraine patients. Considering these findings, it seems like a more appropriate protocol to consider migraine first in patients who describe intense osmophobia and whose diagnosis is doubtful.

In a study of 113 migraine patients, osmophobia was most frequently observed with the smell of perfume, and no significant difference was found between migraine with and without aura groups [15]. Similarly, we found that osmophobia in patients with migraine who participated in our study was most frequent in perfume scents. In patients with TTH, the most common sensitivity was for food odors. Perfumes ranked third among these patients. During patient evaluation, detailed questioning of which odors the patient is sensitive to may support the differential diagnosis. However, it may be useful to warn and inform patients with migraine about osmophobia and smells that may trigger attacks.

In our study, disease duration is the time since the onset of migraine and TTH headaches. Although our study did not show a relationship between disease duration and osmophobia in migraine and TTH patients, there are publications that find a significant relationship, especially in migraine patients. It has been stated that osmophobia is more common in people with long disease duration [6]. It would be appropriate to keep in mind that patients who do not have osmophobia in the first years of migraine diagnosis may develop osmophobia as the duration of the disease increases and to question osmophobia at every examination.

The MIDAS scores of those with osmophobia in the migraine group were slightly greater than those of those without osmophobia, but the difference was not statistically significant. Many studies have shown that the frequency of osmophobia increases as the MIDAS score increases [6,7,16]. We believe that more definitive results can be obtained with studies in which the number of patients is increased.

When age, education level, disease duration, pain frequency, attack duration, and VAS score were examined in both groups of patients, no statistically significant differences were found between any of these parameters and osmophobia. The perception of scents is a subjective experience. Even a patient's current mood can affect their response. For this reason, the results of studies evaluating migraine-triggering odors differ from each other [17].

For the differential diagnosis and treatment of headache, in-depth questioning should be conducted while taking anamnesis, and the characteristics of osmophobia and its accompanying conditions should be questioned. The aim should be to increase the patient's quality of life through strict control by using treatment agents that can cover the patient's comorbidities and reduce osmophobia.

We believe that new studies that divide patient groups into detailed subgroups, provide imaging-supported findings, include a large patient group, and include healthy controls will shed further light on this issue, which we are trying to contribute to with our own series.

Limitations

Our study is retrospective and was studied with a small number of patients. Patients were not divided into subtypes according to migraine classification, and food smells from the odorants were not detailed. Furthermore, migraine and TTH patients were not divided into episodic and chronic subgroups.

## Conclusions

Our study indicates that osmophobia mostly supports migraine as a differential diagnosis in clinical practice. However, osmophobia can be described to a considerable extent in patients with TTH, and it would be useful to use supporting criteria for differential diagnosis and to question the characteristics of osmophobia more deeply. There is a need for further studies on this subject that include different parameters in a large patient series.
